# Does the definition of human ultra-processed foods apply to dog and cat foods? A review of pet food processing techniques, their impact on health, and a call for pet food processing classification

**DOI:** 10.3389/fvets.2026.1690420

**Published:** 2026-03-04

**Authors:** Jirayu Tanprasertsuk, Devon E. Tate, Dominique S. Tarr, Justin Shmalberg

**Affiliations:** 1 Research & Development, KatKin, London, United Kingdom; 2 Department of Comparative, Diagnostic, and Population Medicine, College of Veterinary Medicine, University of Florida, Gainesville, FL, United States

**Keywords:** canine, canned, companion animal, extruded, feline, food processing, NOVA, ultra-processed food

## Abstract

Evidence linking higher intake of ultra-processed foods (UPFs) with increased risk of chronic diseases in humans has grown since the establishment of the NOVA classification and its widespread adoption in nutrition research. Extruded dry and wet diets for cats and dogs (commonly known as kibble and canned/tinned foods) share many features with human UPFs. As these formats are the predominant pet diets in developed countries, similar concerns have been raised. However, progress in this field is constrained by the absence of a clear definition of UPFs in pet nutrition. This Perspective outlines the human UPF definition, with emphasis on the NOVA classification, and reviews mechanisms proposed to explain their negative health impacts in humans. We then compile the current state of knowledge on pet food processing across both traditional dry and wet diets and emerging dietary formats, illustrating the diversity of products available and their potential implications for canine and feline health. We argue that findings from human UPF research cannot be directly extrapolated to dogs and cats, but highlight the need to develop an objective, systematic classification of pet foods based on processing levels. Such a framework would enable research into health effects of processing in companion animals while incorporating the perspectives of manufacturers, regulators, and researchers. Finally, we propose a set of key factors that should inform this classification to reflect the diversity of pet food formats and facilitate its acceptance and use within the veterinary nutrition and pet food research communities.

## Introduction

Food processing includes any home or industrial process that transforms the chemical and/or physical properties of raw ingredients into foods and drinks, aiming to increase food safety, attractiveness, palatability, or shelf life (1). Cooking with heat can improve food safety and palatability, and increase bioavailability of certain nutrients. Industrial food processing has played a significant role in advancing some aspects of public health over the course of its relatively recent history. For example, pasteurisation and canning eliminate pathogenic bacteria, extend shelf life, and make food more accessible through wider distribution (2). Micronutrient fortification has been crucial at the population level in reducing cases of nutrient deficiencies such as birth defects, pellagra, and goitre in humans (3). Certain medical or functional foods, such as baby formula or meal replacement powders, provide essential nutrition to specific populations with unique nutritional requirements or to those that cannot meet their requirements through natural intake alone (4). However, scientific research over recent decades has emerged and suggested that individuals who regularly consume highly processed food and beverage products are at a greater risk of specific diseases compared to those with lower intake levels (5).

## Definition of human ultra-processed foods

The term “ultra-processing” was formally coined recently in 2010 in a peer-reviewed publication by a group of researchers at the University of São Paulo, Brazil (6). Since then, the term “ultra-processed food (UPF)” has become a buzzword in human nutrition research. These researchers established a food classification system based on the level of food processing, known as the NOVA classification (7), and used it as a tool to investigate the relationship between the intake of foods with different levels of processing and various markers of human health. “NOVA” is not an acronym but it is a Portuguese word for “new” chosen to reflect the new classification system when it was introduced. While NOVA is not the only food processing classification system and certainly not a perfect one (8, 9), it provides objective classification (10), and is the most widely used classification in current research, dietary guidelines, and policies (5). NOVA assigns foods and drinks to one of the four groups (Table 1) which are:

- Group 1. Unprocessed or minimally processed foods - foods that are unprocessed or processed with the aim of preserving their integrity, making them suitable for storage, or ensuring they are safe, edible, or pleasant to consume
- Group 2. Processed culinary ingredients - culinary ingredients derived from foods in Group 1 or from nature by processes such as pressing, grinding, milling, and drying, making them suitable for cooking, though not intended to be consumed on their own
- Group 3. Processed foods - foods made by combining items from Group 1 and Group 2
- Group 4. Ultra-processed foods - ready-to-eat, industrially formulated products made mostly or entirely from substances derived from foods and additives, with little to no intact unprocessed or minimally processed ingredients

**Table 1:** NOVA classification [adapted from Monteiro et al. (7) and Martinez-Steele (212)].

Group	Description	Examples of processing	Examples of food and beverage
1	**Unprocessed or minimally processed foods.** Unprocessed foods including edible parts of plants, animals, fungi, algae, and water. Minimally processed foods including natural foods altered by processes to make them suitable for consumption.	Removal of inedible or unwanted parts, drying, crushing, grinding, fractioning, filtering, roasting, boiling, non-alcoholic fermentation, pasteurisation, refrigeration, chilling, freezing, placing in containers and vacuum-packaging, micronutrient fortification and enrichment	Raw or roasted nuts and seeds, fresh or dried fruits, raw green leafy vegetables, herbs and spices, boiled grains and legumes, fortified wheat or corn flour, fresh or dried mushrooms and yeast, boiled eggs, pasteurised milk, meat, poultry, fish and seafood (whole or in the form of steak fillets and other cuts, fresh or chilled or frozen or cooked without added culinary ingredients)
2	**Culinary ingredients.** Substances derived from Group 1 foods or from nature with a purpose to make durable products that are suitable for use in home and restaurant kitchens to prepare, season and cook Group 1 foods.	Pressing, refining, grinding, milling, drying	Cooking oils, butter, lard, sugar, molasses, honey, salt
3	**Processed foods.** Foods or drinks recognisable as modified versions of Group 1 foods made essentially by combining Group 2 and Group 1.	Preservation, cooking, non-alcoholic fermentation	Canned or bottled vegetables and legumes in brine, salted or sugared nuts and seeds, fruits in syrup, salted dried or canned fish, cheeses, freshly made breads, meat, poultry, fish and seafood (cooked by adding only ingredients from Group 2)
4	**Ultra-processed foods.** Formulations made mostly or entirely from substances derived from foods and additives not normally used in culinary preparations, with little if any intact Group 1 food.	Hydrogenation, hydrolyzation, extrusion, moulding, pre-processing for frying	Soft drinks, reconstituted fruit drinks, cereal bars, breakfast cereals, sweet or savoury packaged snacks (crisps, cookies, biscuits), reconstituted meat products, ready-to-eat frozen dishes with additives

In contrast to processed foods (Group 3), which can be manufactured in a factory or prepared at one’s residence, UPFs (Group 4) undergo many industrial processes and often have a long list of ingredients and additives. These are added with the goals of facilitating distribution, extending shelf life, enhancing palatability or convenience (ready-to-eat), lowering manufacturing costs, and optimising mass production. Common industrial ingredients and additives, including examples, are listed in Table 2. Nutritionally, UPFs are often characterised by their high calorie content, low nutrient density, and low cost per calorie (11). UPFs fortified with essential micronutrients or functional ingredients are also common, such as breakfast cereals, cereal bars, packaged bread, fruit drinks, and flavoured dairy products (12, 13).

**Table 2:** Common ingredients and additives in human UPFs and dry and wet pet foods [adapted from Monteiro et al. (7)].

Ingredient	Goal	Examples in human UPFs	Examples in dry and wet pet food*
Ingredients – extracted directly from foods	Used as ingredients	Casein, lactose, whey, gluten	Casein (sodium caseinate), whey, gluten, hydrogenated oils, hydrolysed protein, soy protein isolate or extract, vegetable protein isolate or extract, corn syrup
Ingredients – derived from further processing of food constituents	Used as ingredients	hydrogenated or interesterified oils, hydrolysed proteins, soy protein isolate, maltodextrin, invert sugar, high-fructose corn syrup	

Additive	Goal	Examples in human UPFs	Examples in dry and wet pet food*
Preservatives	Prevent bacterial and fungal growth	Nitrites and nitrates, nisin, sulfites, sodium benzoate, potassium sorbate	Potassium sorbate, rosemary extract
Antioxidants	Prevent oxidation and maintain freshness	Tocopherol, ascorbic acid, erythorbic acid, butylated hydroxyanisole (BHA), tertiary-butyl hydroquinone (TBHQ)	Tocopherol, ascorbic acid, citric acid, ethoxyquin, butylated hydroxyanisole (BHA), butylated hydroxytoluene (BHT)
Stabilisers and emulsifiers	Maintain or enhance the texture, consistency, and chemical and physical characteristics of the final products	Xanthan gum, carrageenan, guar gum, gelatin, pectin, agar, lecithin, carboxymethylcellulose, polysorbate-80	Xanthan gum, carrageenan, guar gum, gelatin, locust bean gum (carob gum), pectin, agar, lecithin, modified starch (corn starch, potato starch, tapioca starch), mono- and diglycerides, glycerol monostearate, sodium caseinate
Natural and artificial dyes	Increase the attractiveness	Beta-carotene, annatto powder, red 40, yellow 5, yellow 6	Beta-carotene, annatto powder, turmeric powder, marigold meal, red 40, yellow 5, yellow 6, blue 2, caramel color
Natural and artificial flavours	Enhance the sensory qualities of foods or to disguise unpalatable aspects of the final product	Methyl anthranilate (grape flavour), isoamyl acetate (banana flavour), cinnamaldehyde (cinnamon flavour), ethyl decadienoate (pear flavour), 2,4-dithiapentane (truffle flavour), benzaldehyde (almond flavour)	Liver flavour, chicken flavour, beef flavour, fish flavour
Flavour enhancers	Enhance the sensory qualities of foods or to disguise unpalatable aspects of the final product	Monosodium glutamate, citric acid, table salt, non-nutritive sweeteners (aspartame, sucralose, sugar alcohols)	Animal digests, animal hydrolysates, animal plasma, citric acid, non-nutritive sweeteners, phosphates (pyrophosphates, phosphate salts)
Carbonating agents	Provide carbonation	Calcium carbonate, sodium bicarbonate	Not common, but monocalcium phosphate and sodium bicarbonate may be used as a leavening agent in dry food
Buffering agents	Control pH, maintain stability and taste	Sodium bicarbonate, citric acid (sodium citrate), phosphoric acid (sodium phosphate), calcium carbonate, potassium carbonate, magnesium carbonate	Sodium bicarbonate, citric acid, phosphoric acid, calcium carbonate, sodium bisulfate
Firming agents	Strengthen the structure and prevent the products from collapsing during processing	Calcium chloride, calcium lactate, monocalcium phosphate	Calcium-based compounds (calcium chloride, dicalcium phosphate, monocalcium phosphate), while serving as firming agents, may be primarily used as sources of calcium fortification
Bulking agents	Increase food volume or weight without changing taste or functionality	Guar gum, psyllium husk, beta glucan, pectin	Guar gum, psyllium husk, beta glucan, pectin, beet pulp, pea fibre, peanut hulls
De-foaming agents	Reduce foaming in food and beverage production	Polydimethylsiloxane, polysorbate, silicon dioxide, polyethylene glycol	Polydimethylsiloxane, silicon dioxide
Anti-caking agents	Prevent clumping and ensure a smooth texture and improve consistency	Powdered cellulose, cornstarch, magnesium stearate, sodium silicate, calcium silicate, magnesium silicate, silicon dioxide, iron ammonium citrate, talc	Powdered cellulose, vegetable fibres, cornstarch, calcium silicate, silicon dioxide, sodium silicoaluminate, sodium bicarbonate, rice hulls, clinoptilolite
Glazing agents	Coat the outside to give a shiny and glossy appearance	Beeswax, candelilla wax, carnauba wax, microcrystalline wax, hydrogenated poly-1-decene, shellac, vegetable oils, animal fats	Carnauba wax, vegetable oils, animal fats
Sequestrants (chelating agents)	Bind to metal ions and prevent fat oxidation or color changes in the foods	Calcium chloride, calcium acetate, sodium gluconate, sodium tripolyphosphate, ethylenediaminetetraacetic acid, tartaric acid	Calcium chloride, trisodium phosphate, sodium tripolyphosphate, ethylenediaminetetraacetic acid, tartaric acid, citric acid, ascorbic acid
Humectants	Keep the products moist and prevent from drying out	Glycerine, honey, sorbitol, lactic acid, propylene glycol, glucose syrup, molasses	Glycerine, lactic acid, propylene glycol, various sugars, corn syrup, molasses, carrageenan, cassia gum, xanthan gum, guar gum

*Examples of listed ingredients and additives can be found on the AAFCO Official Publication and FEDIAF Ingredients or Additives lists.

## Implications of UPFs on human health

Overwhelming epidemiological evidence has emerged showing a correlation between higher portions of UPFs in the habitual diet of humans and increased risks of chronic diseases, including obesity, cardiovascular diseases, cerebrovascular diseases, diabetes, cancers, age-related dementia and Alzheimer’s disease, depression, sarcopenia, frailty, and all-cause mortality (5, 14–22).

Greater intake of UPFs is associated with lower dietary quality (23), higher intakes of added sugars and total fats, and lower intakes of fibre, protein, potassium, zinc, magnesium, and vitamins A, C, D, E, B12, and niacin (24). These nutritional imbalances could contribute to the observed negative impact on health. However, the underlying mechanisms likely involve multiple pathways and are unlikely to be solely due to UPFs’ characteristics of high calories, low nutrient density, and limited amounts of bioactive compounds with antioxidative or anti-inflammatory properties. They are listed below but derived from studies with various degrees of scientific rigour. Supplementary Table 1 lists key studies with meaningful clinical significance in more detail.

- Compared to minimally processed foods, UPFs lead to lower satiety and promote higher calorie intake, which may increase the risk of obesity and other metabolic syndromes in humans (25–28). Short-term habitual exposure to UPFs in healthy-weight individuals also enhances neural responses to food anticipation and consumption, independent of weight gain and changes in insulin resistance (29).
- Extensive food processing leads to deconstruction of the food matrix and the release of acellular compounds (i.e., compounds not contained in cells (30)), which may result in faster nutrient absorption rate and worsened glycemic control, as measured by postprandial plasma glucose and glycated haemoglobin in humans (31, 32).
- Higher UPF intake is associated with an undesirable circulating lipid profile (33, 34) and elevated inflammation (35, 36), both of which are major risks of cardiovascular and cerebrovascular diseases, and metabolic syndromes in humans.
- Rodent experiments show that UPFs increase intestinal microbiome dysbiosis and intestinal barrier permeability, leading to pathogen associated molecular patterns (PAMPs, molecules from pathogens that can be recognised by the host and induce immunological responses), such as lipopolysaccharides, translocating from the gut into the systemic circulation, triggering chronic inflammation (32, 37–39).
- Many UPFs are shelf-stable, meaning that they require sterilisation during manufacturing mainly by moist heat (steam) or dry heat (hot air). For moist heat, temperatures of approximately 121 °C to 129 °C with pressure are used, whereas dry heat requires temperatures from 176 °C to 232 °C for longer duration (40). High-heat processing results in the creation of new compounds such as *trans*-fatty acids and acrylamides, whose intakes have been associated with a range of adverse metabolic outcomes in a few animal and human studies (41). These processes are accelerated as temperatures rise and have important effects at 170 °C and above.
- UPFs are known to have higher levels of advanced glycation end products (AGEs) compared to less processed foods, which are a vast array of compounds generated from mixtures of amino acids and reducing sugars via the non-enzymatic Maillard reaction during food processing at high temperature (42). Higher moisture and lower temperature cooking methods (boiling, steaming, poaching) lead to lower AGE formation than lower moisture and higher temperature cooking methods (roasting, broiling, frying, grilling) (43). Increased intakes of dietary UPFs and AGEs correlate with elevated circulating levels of AGEs in humans (44, 45). Rodent experiments demonstrate that AGEs accumulate in many organs, including the brain, liver, skeletal muscle, and kidneys (46, 47). Both *in vitro* and *in vivo* experiments in rodent models confirm that AGEs bind to the receptor for AGE (RAGE) among other receptors, and induce the cellular oxidative stress signalling cascades that may result in inflammation, fibrosis, or apoptosis, depending on the cell type (48–50). They may interact with other bodily proteins and cause a loss of function. Restriction of dietary AGEs leads to improvements in body fat, insulin sensitivity, circulating lipid profiles, inflammation, vascular function, immune response, and renal function in both humans and mice, with and without diabetes (51–55).
- Even though food additives in human UPFs are regulated by agencies such as the Food and Drug Administration (FDA) in the US and the Food Standards Agency in the UK, Zinocker et al. has compiled a list of studies suggesting that their habitual intake, even at the regulated doses, may negatively impact health (32). For example, emulsifiers have been shown to induce low-grade inflammation and metabolic syndrome by damaging the intestinal mucus barrier and altering the gut microbiome in mice and faecal metabolites in humans (56–58). Similarly, non-caloric and low-calorie sweeteners may bind to receptors along the gastrointestinal tract and change the gut microbiome composition, resulting in immunological and metabolic changes in mice and humans (59–63). Nitrates and nitrites, once consumed and absorbed, are converted to N-nitroso compounds, which increase site-specific cancer risk in humans (64–66).

While UPFs are generally characterised by low nutrient density, some UPFs are fortified or enriched with micronutrients or reformulated with functional ingredients. Little is known about the differential impact of unfortified and fortified UPFs on the overall diet composition and chronic disease-related outcomes (12). One of the researchers who established the NOVA classification has expressed strong opposition to considering fortified or reformulated UPFs as healthy products (67). They argue that the added benefits alone cannot negate the adverse effects inherent to the nature of UPFs.

## Processing in pet food

“Process” is an Association of America Feed Control Officials (AAFCO) Official Feed Term defined as “a method used to prepare, treat, convert or transform materials into feeds or feed ingredients (68).” There is a spectrum of food processing in cat and dog food as in human food production. In many developed countries, the majority of dogs and cats are fed a commercial diet, formulated and supplemented to meet the complete nutritional needs of the animal (69, 70). Standard pet food intake in such countries typically consists of shelf-stable extruded dry and/or retorted wet products (71, 72), which are categorised by moisture content as either dry, semi-moist or wet (73). Dogs are primarily fed dry extruded products, whereas cats are more likely to be fed a combination of dry extruded and retorted wet products (74–77). In recent years there has been a growing diversification of the types of pet food being offered, most of which are also commercially prepared and formulated to meet the complete nutritional needs of the targeted animal.

**Figure 1:**
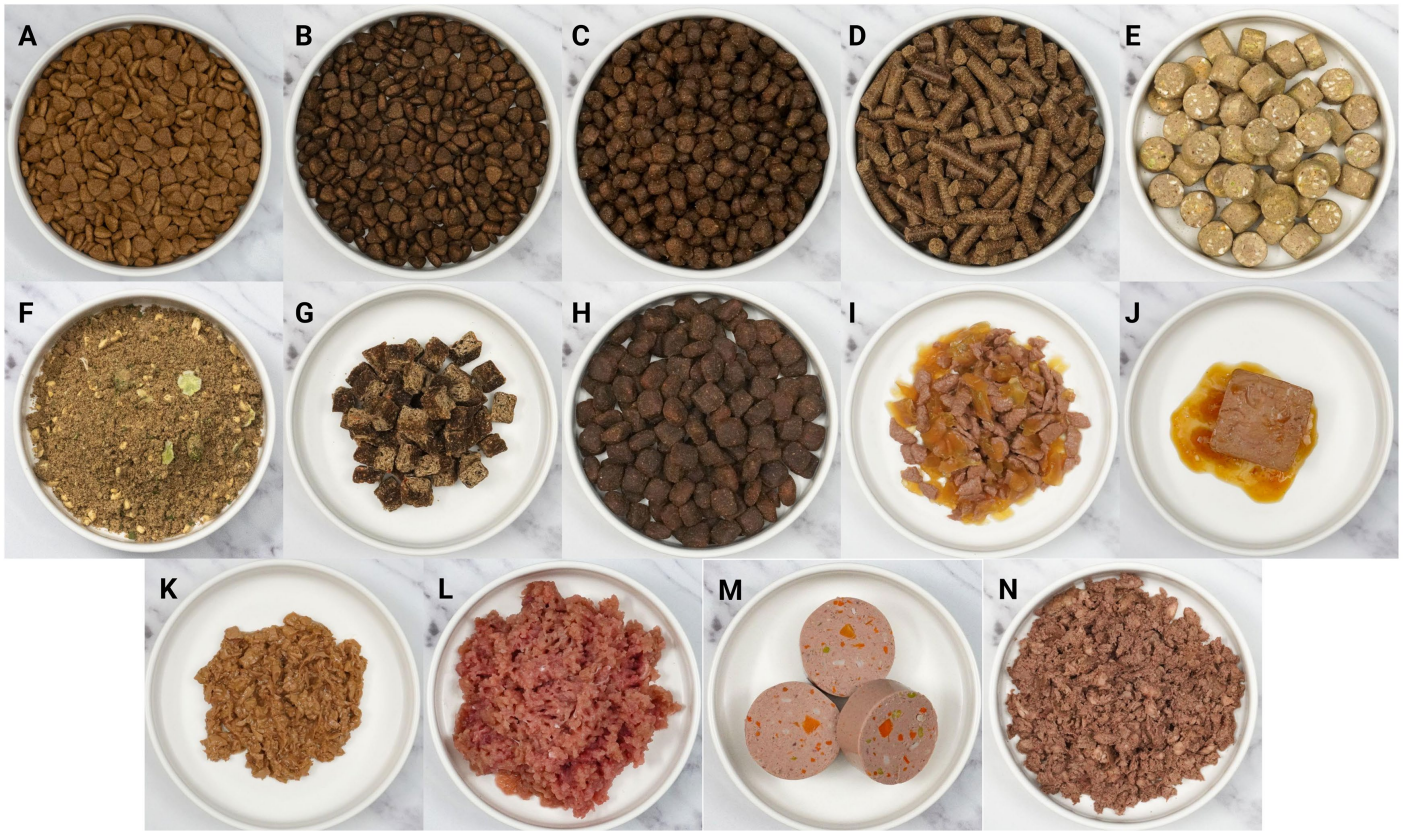
Variety of pet food formats: **(A)** Extruded dry (cat); **(B)** Fresh-included extruded dry (dog); **(C)** Baked (dog); **(D)** Pressed (dog); **(E)** Freeze-dried (dog); **(F)** Dehydrated (dog); **(G)** Air-dried (dog); **(H)** Semi-moist (dog); **(I)** Wet (chunks in gravy, cat); **(J)** Wet (pâté, cat); **(K)** Wet (shreds, cat); **(L)** Raw meat based (dog); **(M)** Mildly cooked, blended (dog); **(N)** Mildly cooked, unblended (cat).

Alternative formats to standard diets (dry/kibble, semi-moist, wet) have not been officially named by regulatory bodies, but are commonly referred to as: fresh-included kibble, pressed, baked, freeze-dried, dehydrated, air-dried, raw meat-based diets (RMBDs), and mildly cooked diets (78, 79). We refer to these formats as “non-traditional” diets henceforth. A description of the various pet food formats available in the market can be found in Table 3, with accompanying images provided in Figure 1. A minimum of 3 products across the UK/EU and USA in each format was reviewed. Whilst information on traditional dry, semi-moist and wet diets is available in appropriate sources (peer reviewed journal articles, published books, patents), very little information is published in professional literature for newer pet food formats. This paper aims to provide a reference description for all pet food formats available, addressing the current absence of a clear, standardised framework in pet nutrition research. There are, in some cases, related industry definitions that need to be better reconciled to fit a processing framework. As an example, mildly cooked diets describing non-shelf-stable products treated with a level of thermal processing are commonly marketed as “fresh” diets in industry and marketing; however, a regulatory definition of fresh exists only for ingredients rather than finished diets. A recent review also shows that the definitions of RMBDs are highly subjective and rely on the interpretation of pet food manufacturers, researchers, consumers, and animal food regulatory authorities (80). The lack of appropriate published information on and definitions of alternative pet food formats limits academic discussion and research into these formats and highlights a need for this gap to be filled.

**Table 3:** Pet food formats available on the market.

Proposed category name	Figure	Categorical definition	Marketing names	Ingredient List of the Sample Product
Extruded dry pet food	Figures 1A,B	A process by which ingredients, including meat meals (traditionally rendered) and a source of starch (typically grains) are ground, mixed and passed through a feeder into a pre-conditioner where continuous mixing and the addition of moisture in the form of water or steam takes place; with the temperature of the mix rising to 70–90 °C – partially cooking and plasticizing the mixture. The mixture is passed into the extruder, containing an internal screw that drives the creation of friction, which, with other thermal sources (such as steam injection or electrical heating of the surrounding barrel), increases the temperature of the mixture in the range of 125–150 °C for 10–270 s, further cooking the ingredients, gelatinizing the starch and forming a dough. The dough exits the extruder through a die and face cutter, forming the kibble shapes and landing on a conveyor belt that passes through a drier where the pieces are dried by heated air at 90–180 °C for either single or multiple passes. Still warm, kibbles are then coated by a sprayed-on mixture of liquid fats, palatants and vitamins, reaching a final moisture content of <12% and producing small, typically brown, crunchy pieces (212–214).	Kibble Biscuits	Figure 1A (cat) – chicken (including bone, meat, skin), dried poultry protein, wheat, corn, soya meal, wheat gluten, animal fats, corn protein meal, dried chicory root, corn grits, nutritional additives, digest (with added heat treated *Lactobacillus Delbrueckii* and Fermentum powder), dried yeast, antioxidants Figure 1B (dog) – chicken (including freshly prepared chicken, dried chicken, chicken stock), sweet potato, peas, potato, beet pulp, linseed, omega-3, nutritional additives, vegetable stock, marjoram, basil, oregano, sage, thyme, parsley, fructooligosaccharides, mannan oligosaccharides
Baked pet food	Figure 1C	A process by which ingredients, including meat meals and/or meats and flours (typically pre-gelatinized starches due to lower processing temperatures), are initially ground, mixed and hydrated to a moisture content of approximately 33–35%. The mixture is kneaded into a dough and shaped using a rotary moulding or manual cutting, forming uniform pieces. The pieces are passed through a tunnel or convection oven where baking takes place by thermal conduction, convection and radiation at temperatures typically ranging from 175–230 °C for a period of 10–25 min. The pieces are cooled to ambient temperatures and may be coated with fats and palatants. The final pieces are small, brown and crunchy, although denser than kibble, with a typical moisture content of 10% or less (215, 216).	Oven-baked Biscuits	Figure 1C (dog) – chicken (dried chicken, fresh chicken), dried sweet potato, dried peas, dried beet pulp, chicken fat, dried alfalfa, chicken gravy, mannan oligosaccharides, chicory root extract, dried botanicals (dandelion leaf, marigold), yucca extract, nutritional additives
Pressed pet food	Figure 1D	A process by which ingredients, typically pre-cooked or dried, are ground into small bits and mixed with water and/or fat. The mixture is then passed through a pellet mill where it is compacted or moulded by pressure and heated by steam up to 80 °C. The mixture is pushed through die openings and cut at the desired length, producing a smooth rod shape, and cooled to ambient temperature with a final moisture content of around 10% (216).	Pelleted Cold-pressed	Figure 1D (dog) – chicken (including freshly prepared chicken and dried ground chicken), dried sweet potato, dried peas, poultry fat, gelatin, dried beet pulp, dried fruit (apples, pears, blueberries, cranberries), salmon oil, brewer’s yeast, dried botanicals (fennel, nettle, dandelion), mannan oligosaccharide, egg powder, chicory extract, dried seaweed, camomile, flaxseed oil, glucosamine, chondroitin sulphate, yucca extract, nutritional additives
Freeze-dried pet food	Figure 1E	A process in which raw ingredients are weighed, ground and blended or emulsified. The mixture can undergo high-pressure processing – a non-thermal treatment that inactivates microorganisms and spores at extreme temperatures of 100–1000 MPa, with or without heat (217). The mixture is then frozen, crystallising water molecules, followed by sublimation – the primary drying step, where ice is directly converted to vapour under pressure and heat. This is followed by a second drying step, the desorption phase, using lower pressure and heat to remove the final remaining moisture. Shelf temperatures of up to 70 °C can be used. Light coloured pieces with a moisture content of around 2% are produced (218, 219).		Figure 1E (dog) – chicken, potato, sweet potato, peas, carrot, apple, pear, nutritional additives, technological additives (antioxidants)
Dehydrated pet food	Figure 1F	A process where ingredients are mixed, laid out on trays on stationary racks in an oven where warm air, around 70 °C, circulates over an extended period of time which can be several hours. The resulting product can be muesli or nugget-like pieces, has a moisture content of around 10% and is often intended to be rehydrated.		Figure 1F (dog) – chicken, potato, dried egg, peas, chicken stock, carrot, linseed, broccoli, spinach, nutritional additives, omega-3, strawberries, chicory extract, glucosamine, chondroitin, beta carotene
Air-dried pet food	Figure 1G	A process in which ingredients are ground, mixed and dried on stationary racks or a conveyor belt in an oven where warm air circulates over an extended period of typically several hours or more at temperatures around 50–70 °C. The resulting products are typically jerky-like pieces with a final moisture content of around 10–14% (81, 220, 221).		Figure 1G (dog) – chicken, dried sweet potato, rapeseed glycerine, dried potato, minerals, dried carrot, dried thyme, dried chicory root, fibre, beet pulp, nutritional additives
Semi-moist pet food	Figure 1H	A process where ingredients are ground, mixed and passed through the feeder into the pre-conditioner where continuous mixing takes place and moisture is added in the form of water or steam, raising the temperature of the mix to around 60 °C. Other liquids and flavours can be added directly into the preconditioner during this stage. The mixture is then passed into the extruder, containing an internal screw that drives the creation of friction, converting into mechanical energy, which, with other thermal sources (such as steam injection or electrical heating of the surrounding barrel), increases the temperature of the mixture to cook the ingredients, gelatinize the starch, and form a dough. Differently to extruded kibble, the mixture is heated at temperatures below 100 °C and then cooled in the last portion of the extruder to form minimum expanded products. The dough exits the extruder through a die and face cutter, forming the pieces, which are then mixed with water, humectants and acids in coating drums, producing small, brown pieces with a moisture content of 15–50% (81, 222).		Figure 1H (dog) – fresh chicken, sweet potato (dried), poultry protein (dried), potato (dried), protein hydrolysate, peas, chicken liver, salmon oil, poultry fat, forest berries (dried: lingon berries, blueberries, elderberries), herbs (dried: stinging nettle, blackberry leaves, common yarrow, fennel, caraway, chamomile, mistletoe leaves, gentian root, centaury), yeast (mannan oligosaccharides, beta-glucans), sunflower oil, safflower oil, chicory (dried, source of inulin), nutritional additives, technological additives (antioxidants)
Wet pet food	Figure 1I – chunks, Figure 1J – pâté, Figure 1K – shreds	A process where animal proteins are first ground to minimise particle size. For loaf-style products, ground animal source ingredients are homogenised and heated to coagulate proteins and create a semi-solid mass before packaging (223–225). For chunk-based formats, restructured meat pieces are typically prepared by emulsifying animal proteins in a bowl chopper with a heat settable component, such as blood plasma, before being set by steam (above 70 °C, ideally 70–105 °C). Otherwise cheaper meat analogues can be used, substituting animal protein with non-animal protein such as wheat gluten or soy protein and processing at higher temperatures (at least 110 °C, can be higher at 130–240 °C, to coagulate non animal proteins), followed by extrusion. Restructured meat and meat analogue slabs are then chopped or shredded and combined with gravy/sauce/jelly and packaged (17, 18). Packaged products are finally sterilised through retort processing under heat (115–130 °C for 20–120 min) and steam pressure to ensure shelf stability (14, 17). The final product typically has a moisture content of around 80% and consists of homogenous meaty pieces or a loaf.	Canned Tinned Pouched Moist Soft	Figure 1I (cat) – meat and animal derivatives (including chicken in the chunk), cereals, derivatives of vegetable origin, minerals, various sugars, nutritional additives, sensory additives (flavourings) Figure 1J (cat) – meat and animal derivatives (including chicken), derivatives of vegetable origin, minerals, various sugars, nutritional additives Figure 1K (cat) – meat and animal derivatives (including chicken), vegetable protein extracts, fish and fish derivatives, minerals, derivatives of vegetable origin, various sugars, nutritional additives, flavourings
Raw meat based diets (RMBDs)	Figure 1L	A process where raw meat, offal, and/or by-products in frozen blocks are broken up, mixed with other ingredients and ground. The mixture is then filled into its packaging, vacuum sealed, and frozen (226). High-pressure processing or the spraying of bacteriophages onto the product are optional steps that can be applied for food safety (227, 228). The final product typically has a moisture content of around 65–75% and appears as minced raw meat (often with vegetables).	Biologically appropriate raw food	Figure 1L (dog) – chicken carcass, chicken trim, chicken heart, chicken liver
Mildly cooked diets	Figures 1M,N	A process where raw ingredients are minced or ground, mixed, packaged, and then cooked by either steaming in chub format (Figure 1M), or kettle cooking or steaming (Figure 1N). Alternatively, ingredients can be cooked separately before being blended together and packaged. These diets are typically cooked at the lowest temperature required by legislation for food safety, around 70–90 °C. The final product typically has a moisture content of around 65–75% and appears as cooked meat (often with vegetables).	Fresh diets Frozen fresh	Figure 1M (dog) – chicken, chicken liver, potatoes, pea fibre, egg, spinach, garlic powder, celery powder, nutritional additives Figure 1N (cat) – fresh chicken (thigh, liver, heart), nutritional additives

* “meat” includes fish.

Traditional dry pet food primarily uses rendered meat meals as a concentrated source of protein. While traditional wet pet food formats typically rely on mixtures of water, animal tissues (including organ meats), fats, vegetable ingredients (including grains), vitamins, and minerals, often combined with emulsifiers or gelling agents to achieve the desired texture (81). In contrast, newer and non-traditional pet food formats are often positioned as opting for (or increasing the inclusion of) less processed ingredients, using processing methods marketed as involving reduced heat treatment, and often involving fewer processing steps; however, published research characterising and directly comparing these processes and products remains limited. Whilst rendered meat meals may still be incorporated into some newer or non-traditional pet food formats (e.g., fresh-included kibble, baked, semi-moist, pressed), these products typically differentiate themselves through the inclusion of freshly prepared meat, alternative grains and starches, botanicals, herbs, bioactives, and functional ingredients. Other non-traditional formats of pet food (e.g., freeze-dried, air-dried, RMBDs, mildly cooked diets) index heavily on freshly prepared or whole meat and often include vegetables, fruits, herbs, and botanicals. Many of these products use human grade ingredients and processing methods with low or no heat treatment, and have simpler production processes (with the exception of freeze-dried). Additionally, RMBDs and mildly cooked diets also typically exclude the use of emulsifying, thickening and gelling agents commonly used in traditional wet pet food production (e.g., carrageenan, cassia gum, wheat gluten). Within the wet pet food category there are also trends towards using whole meats instead of reconstituted meats, and thickeners such as tapioca starch, that are better perceived by customers. These categories and trends align with pet owners’ increasing emphasis on food safety, quality, traceability, processing transparency, and health benefits, driven by the growing humanisation of pets. However, stronger evidence of these attributes would be required to substantiate the differentiation of these categories (82).

It is evident that a simple categorisation of pet food into dry, semi-moist, and wet is no longer representative of the wide variety of pet food processing techniques and ingredients that are available. Ingredients and degree of processing can vary greatly between formats that would historically both be categorised as “dry” or “wet.” Adding to this complexity is the distinction between regulatory terms (defined in AAFCO) such as “human grade” and “feed grade.” These designations refer to differences in handling, storage, and facility compliance requirements, rather than inherent ingredient quality. However, a direct comparison assessing the difference in quality in human grade and feed grade ingredients has not been conducted. These regulatory pathways may also differ in food-safety risk, as illustrated by documented pentobarbital contamination events in rendered pet food ingredients (83, 84). Although food safety standards have since changed, follow up data providing evidence of closer alignment between feed and human grade ingredients has not been conducted. Recent innovations, such as lab-grown or cultivated meat, produced by harvested cells of a living animal or egg in a bioreactor, further complicate distinctions in pet food processing and ingredient categorisation (85, 86). RMBDs and mildly cooked diets can also be either commercial or home-prepared, with home-prepared versions often carrying a greater risk of nutritional deficiencies (87), making it difficult to define these diet types consistently in terms of processing and quality control. Furthermore, our reference nutrient values are based on semi-purified or purified diets that were often extruded, while potential differences across formats need to be addressed to ensure accuracy. Changes to raw materials to meet nutrient recommendations could also introduce complications, such as ongoing debate over chelated copper use and the lack of an AAFCO maximum for copper in feed (88). A revised approach to standardising pet food classification will assist further research into the advantages and disadvantages across categories, which will ultimately aid more informed discussions between the veterinarian and pet owner when selecting the right food to support their pet’s optimal health (71).

## The definition of human UPFs cannot be applied 1:1 to pet food

Similarities and differences between human UPFs and traditional dry and wet pet diets are listed in Table 4. Examples of common ingredients and additives in dry and wet pet foods are also listed in Table 2. Given the extensive processing and additives involved in producing extruded dry kibble and canned pet food, one may mistakenly adopt the human food classification such as the NOVA and equate these pet foods to UPFs. However, the definition of human UPFs from the NOVA classification cannot be directly applied to pet food. Unless it is labelled for “intermittent or supplemental feeding only” or to be used under direct supervision of a veterinarian, commercial pet foods are nutritionally complete and must provide all essential nutrients required for growth or maintenance and meet the minimum requirements for each life stage (71). This can be achieved through the use of various natural ingredients during formulation or through fortification. Such regulations are strictly enforced by governmental agencies using guidance from organisations such as European Pet Food Industry Federation (FEDIAF) in the UK and the EU, and AAFCO in the US. Meanwhile, there is no similar requirement for human UPFs which are often characterised by low nutrient density (11).

**Table 4:** Similarities and differences between human UPFs and traditional dry and wet pet foods.

Features of human UPFs	Features of traditional dry and wet pet foods
Shelf-stable UPFs are sterilised at high temperatures (>100 °C) and/or pressures. Processing may include extrusion or canning.	Dry and wet pet foods are shelf stable and sterilised at high temperatures (>100 °C) and/or pressures. Dry food is extruded whereas wet food is canned or sterilised in a tray or a pouch.
UPFs use industrial grade ingredients and include ingredients that are extracted directly from foods or derived from further processing of food constituents.	Most dry and wet pet foods use feed grade ingredients and include ingredients that are extracted directly from foods or derived from further processing of food constituents.
Use of food additives and industrial processing are designed to create durable, shelf-stable, accessible, convenient, attractive, palatable products for consumers.	Use of food additives and industrial processing are designed to create durable, shelf-stable, accessible, convenient, attractive, palatable products for pets and/or pet owners. Many additives may however confer nutritive value or have other positive impacts on health.
Greater intake of UPFs is associated with lower dietary quality, higher intakes of added sugars and total fats, and lower intakes of fibre, protein, potassium, zinc, magnesium, and vitamins A, C, D, E, B12, and niacin.	A nutritionally complete commercial pet food must provide all essential nutrients required for growth or maintenance and meet the minimum requirements for each life stage. However, diets labelled for “intermittent or supplemental feeding only” or designed to be used under direct veterinary supervision and treats do not need to be nutritionally complete.

The definition and scope of ingredients and additives are also different in human food and pet food. In the US, Code of Federal Regulations Title 21 (21 CFR) Subchapter B lists ingredients and additives suitable for human consumption (89). AAFCO Official Publication Chapter Six provides standardised definitions and terms for ingredients and additives in animal feed. It lists “additive” as an Official Feed Term defined as “an ingredient or combination of ingredients added to the basic feed mix or parts thereof to fulfil a specific need. Usually used in micro quantities and requires careful handling and mixing (68).” Likewise, the UK and EU regulations provide a list of feed materials (Commission Regulation (EU) 68/2013 and 2017/1017) (90, 91), and permitted feed additives for use (Regulation (EC) 1831/2003) (92). EC 1831/2003 groups feed additives into five categories which are technological (preservatives, antioxidants, emulsifiers, stabilisers, thickeners, gelling agents, binders, substances for control of radionuclide contamination, anticaking agents, acidity regulators, silage additives, denaturants), sensory (colourants, flavouring compounds), nutritional (vitamins, compounds of trace elements, amino acids, urea derivatives), zootechnical (digestibility enhancers, prebiotics, probiotics), and coccidiostats & histomonostats (anti-parasitic substances).

The supply chain for pet food and human food also differs in several ways. Pet food can include ingredients not fit for human consumption, and by definition, once an ingredient is converted to a pet application it is no longer considered fit for human consumption. Unless indicated as human grade in countries such as the US which has adopted AAFCO regulatory definitions of “human grade,” pet food manufacturing facilities have different regulations for sanitation, personnel training, food handling, storage, and processing than human food manufacturing facilities. Therefore, many ingredients and ingredient sources found in pet food are not used in human food. Likewise, ingredients and additives that are deemed safe for human consumption, but are toxic to pets at dietary doses, are also excluded from commercial pet foods (93).

## Implications for pet health

Based on the rationale that: (1) pets and humans have different physiologies and nutrient requirements; (2) certain ingredients, raw materials, and food additives are used in pet foods but not human foods, and vice versa; and (3) the NOVA classification and the definition of human UPFs cannot be directly applied to pet food, the negative health impact of UPFs observed in humans should not be directly extrapolated to pets.

Although diet type is frequently implicated as a risk factor for several feline and canine diseases (94–97), there remains a significant gap in research assessing the health impacts of different types of pet foods and the consequences of food processing methods. Furthermore, most published comparisons are limited to a small number of specific commercial formulations within each diet format, and therefore may not be fully representative of the diversity of products available. Without well-designed randomised controlled trials incorporating a wider range of brands and formats, the causal relationship between diet types and disease risk in pets remains ambiguous, and it cannot be conclusively stated that one diet type is healthier than another. It is also essential to note that current studies comparing dietary formats cannot fully disentangle the interactive effects of moisture content, energy density, nutrient concentration, and ingredient composition. Observed health outcomes likely reflect the combined influence of these factors rather than processing alone. That said, emerging evidence, outlined in the subsections below, suggests differential processing methods may influence health in dogs and cats, but such findings should be interpreted with caution until studies are conducted that control for these confounding factors.

### Weight and metabolic health

Risk of feline obesity has been reported more frequently among cats consuming dry diets (98–100), likely due to the combined influence of their low moisture content, high energy density, inaccurate portioning, and the tendency for cats to over consume to reach satiety (98). Conversely, increased inclusion of commercial wet food has been associated with a lower risk of overweightness in cats (99, 100), potentially due to its higher moisture content and lower energy density (101). While similar associations between diet type and obesity risk have not been consistently found in dogs (102–104), lower-cost diets have been implicated as a potential risk factor (105, 106). Moreover, weight outcomes are likely influenced by feeding practices, as dog owners have been shown to consistently overestimate dry food portions (107). In both species, excessive energy intake remains the primary driver of obesity, highlighting the importance of portion control particularly with low-moisture, high energy density diets.

In a therapeutic context, both dry and wet commercial weight-loss diets have been shown to effectively promote weight loss in overweight pets (108–114). These diets are typically formulated to be higher in protein and lower in fat, with higher nutrient:energy ratios often with elevated fibre content (113–116). However, research indicates that while these diets are generally effective and support nutritional adequacy during restriction, their success with respect to weight loss primarily reflects controlled caloric intake rather than processing method or formulation, with comparable outcomes observed when energy restriction is applied to non-therapeutic diet formats (117–120). Given current evidence, the relationship between food processing and obesity risk in pets remains unclear. Overall, obesity outcomes in pets appear to be influenced more strongly by factors such as water content (in cats), caloric density, portioning, and formulation, while the impact of diets from emerging categories remain unclear.

### Oral health and microbiome

Commercial dry diets have been associated with improved oral health in pets compared to home-prepared, wet, or soft diets, even after controlling for age (121–123). Evidence indicates that dry diets may promote greater mechanical abrasion than softer diets, a primary factor in reducing plaque accumulation, tartar buildup, and periodontal disease. Cats fed dry diets also exhibited increased diversity of the oral microbiome than cats consuming wet diets (canned, sachet and/or fresh meat combinations) (124), although the health implications of this remain uncertain. Similarly, in dogs, a dry diet was associated with a higher abundance of oral health-associated bacterial taxa, along with reduced breath odour and plaque buildup, compared to dogs fed a wet canned diet (125). Beyond standard dry diets, specialised dental care kibbles, formulated and processed to be larger in size with a porous texture, are designed to allow tooth penetration, thereby promoting increased mastication and abrasion, which has been shown to modestly aid in the reduction of plaque and tartar buildup in pets (126–128). While current evidence supports some oral health benefits of certain dry diets in pets, particularly specialised kibbles, these effects are modest and primarily attributable to their physical properties. Such benefits are not universal and should not be regarded as a substitute for regular dental hygiene measures. Additionally, with limited research on oral health outcomes in pets fed non-traditional diets, further investigation is needed before drawing definitive conclusions.

### Digestive health and gut microbiome

Longitudinal and cross-sectional questionnaire-based studies have indicated that feeding puppies and adolescent dogs RMBDs reduces the incidence of chronic enteropathy in adulthood, whereas commercial dry diets were associated with a higher incidence (96, 129). However, it is important to note that questionnaire-based studies are prone to bias and confounding. Similarly, RMBDs and mildly cooked meat-based diets have also been linked to improved faecal outcomes, including firmer faecal scores and reduced faecal outputs, compared to commercial dry or wet diets (130–136). However, there’s variability based on the specific diet composition and the individual pet. While these studies suggest non-traditional processing methods may offer a protective effect, these diets also have inherent differences in their ingredient and macronutrient profiles. Notably, dry and wet therapeutic gastrointestinal diets, formulated to be highly digestible, with tailored fatty acid inclusions and dietary fibre levels, have also been effective in managing chronic gastroenteropathies in cats and dogs (137–141).

Differences in faecal microbiota composition have also been found between traditional and emerging diet formats in pets (133, 134, 136, 142–145), suggesting that diet processing may play a role in shaping the gut microbiome. However, these effects may also result from differences in ingredient profiles, nutrient content, particle size, gut transit time, and nutrient digestibility. In addition, controlled studies using identically formulated diets subjected to different shear extrusion intensities have demonstrated that processing conditions alone can alter microbial redox balance, short-chain fatty acid production, and immune responses in dogs and cats (146–148). While the long-term health implications of these gut microbial shifts remains unknown, particularly as the majority of studies have included animals absent of any major diseases, these findings strengthen the evidence that food processing parameters may impact digestive and microbiome health in pets.

### Nutrition and digestibility

Nutritional imbalances have been reported in both commercial and home-prepared RMBDs (149, 150), yet commercial dry and canned foods also experience food recalls due to nutritional imbalances (71). Studies assessing apparent nutrient digestibility have reported higher digestibility values for non-traditionally processed diets compared with traditionally processed commercial dry or wet diets in cats and dogs (130, 131, 133, 135, 136, 145, 151). While high digestibility promotes nutrient absorption, it does not necessarily ensure nutrient bioavailability, and its broader implications for long-term health in pets remains unclear. However, in certain conditions such as obesity, the inclusion of less digestible ingredients, particularly fermentable and non-fermentable fibres, can aid in energy management by promoting satiety and modulating nutrient absorption, thereby helping to control caloric intake when owner regulation is insufficient (94, 152).

### General health and disease management

Emerging evidence suggests potential benefits of non-traditionally processed diets for pets, although the majority of benefits are based on non-specific health studies and correlations in dogs and cats (149, 150). Client-owned dogs fed a RMBD showed an improvement in clinically evaluated health scores compared to dogs fed a commercial extruded diet (153). While clinical scoring was performed by a blinded veterinarian, owner bias and placebo effects cannot be fully ruled out. Similarly, improved overall health scores have been observed in healthy household dogs transitioning from an extruded diet to a mildly cooked diet in a single-arm trial (134). After 4 weeks, 61% of owners reported better overall health, 48% noted improved coat condition, and 48% observed higher activity, with no reports of worsened outcomes. However, these owner-reported improvements may reflect perceived rather than objective changes. In healthy Ragdoll cats, cooked and RMBDs not only resulted in higher digestibility, but were associated with improved fur condition, faecal scores, serum antioxidant capacity, and immunity compared to an extruded dry diet. The cooked diet yielded the most favourable outcomes overall, although macronutrient differences were present between the extruded diet versus the cooked and RMBDs (133). Additionally, dogs fed a RMBD exhibited lower levels of urinary oxalate and calcium than those on a dry diet. These changes, likely driven by a combination of lower dietary oxalate and starch content, reduced sodium and calcium intake, increased moisture, and differences in nutrient bioavailability, may reduce the risk of urolithiasis in predisposed dogs (154). Differences in immunological and inflammatory signaling have also been observed between mildly cooked and dry diets in dogs. In a crossover study with healthy client-owned dogs, a mildly cooked diet led to a lower TNF-*α*-to-IL-10 ratio and higher IL-8 production compared to extruded dry diets (155). While no differences were detected in remaining inflammatory markers between diet types, these changes suggest potential immunomodulatory and anti-inflammatory effects with a mildly cooked diet. Further research is needed in diseased dogs, as well as to determine whether these effects arise from specific ingredients, processing characteristics, or interactions between multiple components of the mildly cooked diet.

At the same time, commercially available therapeutic dry and wet diets are formulated with precise nutrient profiles or textures to benefit specific medical conditions in pets. For example, specialised therapeutic formulas enriched with essential fatty acids and targeted nutraceuticals have been found to support joint health (156–159), dermatological conditions (160–162), and cognitive function in pets (163, 164). Similarly, renal diets formulated with reduced phosphorus and enhanced antioxidant properties, help slow the progression of renal diseases in cats and dogs (165–169). These diets are traditionally processed, and their health benefits are well documented. However, most studies evaluating these diets are pre-post studies (lacking a comparator) or compared against diets using the same processing methods. To truly understand the impact of food processing on specific diseases, traditional therapeutic diets need to be evaluated against diets with different processing methods in pets with the same disease or symptom, while controlling for ingredients, nutrient content, and intake.

### Contamination, safety, and processing concerns

A common concern with RMBDs is pathogenic contamination due to the lack of heat processing. Commercial RMBDs, particularly those not subjected to a post-processing step like high-pressure pasteurisation, have been found to have a higher risk of contamination with pathogenic organisms than commercial dry and wet pet foods, which has been indicated as a health risk to both pets and humans (71, 149, 150, 170, 171). Recent outbreaks of highly pathogenic avian influenza have further highlighted concerns about RMBDs or unpasteurised animal products in pet foods, prompting stricter sourcing regulations and monitoring. Kittens fed a RMBD have also been observed to shed higher levels of pathogenic organisms in their stool (132), which may increase environmental exposure and potential transmission to people. Mildly cooked diets may offer a safer alternative for those seeking non-traditional diet formats without the risks associated with RMBD feeding. However, contamination risks are not exclusive to RMBDs. Recalls on traditional dry and wet commercial foods are known to occur for various reasons, largely driven by the quality and safety of the ingredients. These include chemical contamination likely introduced through the ingredient supply chain (172), toxic metals exceeding maximum tolerable levels (173, 174), and aflatoxin contamination, a common mycotoxin in grains that has caused severe illness and death in dogs (71). Additionally, *Salmonella* contamination as a result of post-processing handling and use of rendered animal products and/or palatants (175). However, recall frequency is influenced by production volume, with commercial dry and wet pet foods accounting for the largest market shares and therefore would be expected to account for a greater number of recalls overall.

Beyond contamination risks, traditionally processed pet foods also contain biogenic amines and AGEs as a result of ingredient sources, cooking at high temperatures, or inappropriate storage. Biogenic amines and AGEs have known mutagenic, cytotoxic, and pro-inflammatory effects, and have been hypothesised by some researchers to be associated with cancer and other age-related diseases in pets even at the concentrations found in pet foods (176–181). A study comparing dietary and plasma concentrations of AGEs in canned wet food, dry food, air-dried food, and mildly cooked food found that commercial wet dog food had the highest levels of dietary and plasma AGEs (182). Similarly, dogs fed a fresh diet suspected to be minimally cooked, though exact processing parameters were not disclosed, demonstrated reduced serum AGE levels and enhanced markers of mitochondrial and fatty acid metabolism compared with an extruded kibble; however, differences in macronutrient composition between the diets may have confounded these findings (183). While lower-temperature processing reduces AGE formation, concentrations can still vary significantly based on factors such as ingredient composition, particularly protein and sugar content, as well as moisture levels and overall processing conditions (43, 184). However, the long-term impact of AGEs on pet health remains an area for further research.

Additionally, traditionally processed pet foods often include additives to enhance stability, sensory appeal, and nutritional value (185). While only approved additives are permitted in pet foods, the safety of many remains insufficiently studied in dogs and cats. Some additives, though deemed effective for technological purposes, have been evaluated without species-specific data, and existing assessments often highlight gaps in toxicological evidence and uncertainties in long-term safety (185–187). For example, propylene glycol, a synthetic antimicrobial preservative and humectant, has been shown to cause haematological abnormalities in cats at high doses in controlled studies (185), prompting its prohibition in cat food by the US FDA. However, despite these findings, it remains permitted in semi-moist dog foods and treats, and appears not to be explicitly prohibited in cat food in the EU, where it is classified as a feed material (185). Synthetic antioxidant preservatives such as ethoxyquin and sulphites have been the subject of regulatory scrutiny. While robust evidence of harm in pets is limited, a metabolite of ethoxyquin has been identified as possibly genotoxic, and an associated impurity as potentially mutagenic, leading to its withdrawal as a feed additive in the EU (185). Similarly, sulphites have been linked to thiamine deficiency in dogs and cats in case reports and studies, prompting mandatory thiamine supplementation in sulphite-containing products in Australia (185). These concerns are based on precautionary regulatory actions rather than definitive evidence of widespread toxicity. Dietary guar gum, a gelling agent, reduced protein digestibility and faecal quality when added to a canned cat food, particularly in senior cats (185). Negative impacts on faecal output have also been reported in dogs, but these effects appear to relate to ingredient characteristics and are dose dependent (185). An impurity in cassia gum, a widely used gelling agent, has been flagged as potentially carcinogenic for dogs and cats, prompting its restriction to purified forms in the EU (185). Taken together, these examples highlight the importance of rigorous, species-specific safety testing for additives used in pet foods.

### Overall considerations

The relationship between food processing and pet health is complex, with both traditionally and non-traditionally processed diets presenting potential benefits and risks. While non-traditionally processed diets are gaining popularity for their perceived health advantages, they remain understudied. In contrast, while traditionally processed diets have been more extensively studied, the diversity of the formulations, ingredients, and specific processing methods included within this diet format further convolutes the relationship. Overall, considering that some diets can be commercially manufactured or home-prepared, and may incorporate a wide range of ingredients and nutrient formulations, it is difficult to generalise these results with the current state of evidence. Given these intrinsic interdependencies, the relative contribution of processing to health outcomes cannot yet be quantified, and further research with properly designed studies is needed to better understand the isolated impact of food processing methods on pet health. Until such data are available, conclusions about the health implications of specific diet formats should remain tentative, and dietary choices should be guided by the pet’s individual needs, with attention to portion control, nutrient balance, and health status.

## A call for a pet food classification and more research

As previously mentioned, a consistent association between greater intakes of UPFs and poorer health outcomes are observed in humans. The findings in human nutrition research cannot be directly extrapolated to dogs and cats, due to inter-species differences in physiology, nutrition requirements, and the included ingredients, raw materials, and food additives in the food supply (not only between humans and pets, but also between dogs and cats). Even with an attempt, a similar examination cannot be performed in pets since currently no systematic classification based on processing level exists, and the diversification of pet food formats is making it increasingly unclear how they map onto the spectrum of food processing. This lack of a consistent framework hinders research and makes it challenging for pet owners and veterinary professionals to objectively compare and evaluate different diets and their impact on health outcomes. Recent discussions and debates about processing and category definitions outside of academic journals demonstrate the need for a standard framework to disclose processing variables in scientific studies. Inspired by the NOVA classification system used in human nutrition, we propose a list of factors that should be considered to be included in a classification system for pet foods to capture the complexity of modern pet food manufacturing. This initial framework aims to highlight the diversity and complexity of pet food processing, provide a more nuanced understanding of pet food characteristics, and facilitate more robust research in the field. This processing-based classification is not intended to replace the current industry-standard moisture-based categories (dry, semi-moist, wet), but rather to operate alongside existing industry terminology. The authors also encourage that an expert panel consisting of stakeholders in the pet food industry such as food manufacturers, regulatory officers, nutritionists, researchers, and clinicians should be assembled to initiate a roundtable discussion on this particular topic, since much research, data, and debate, are still needed to establish a practical and comprehensive classification.

**Figure 2:**
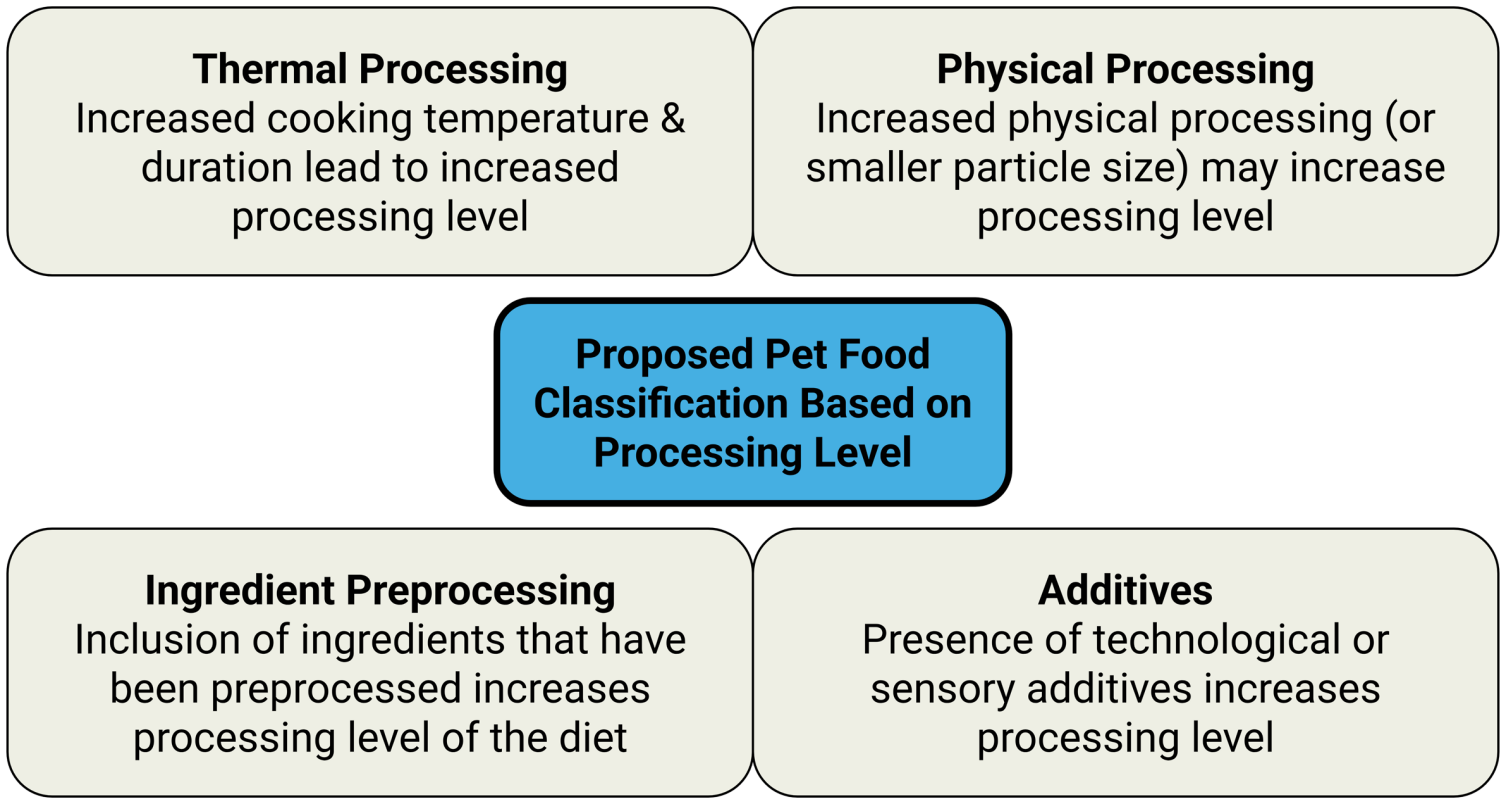
Proposed pet food classification based on processing level.

We propose that the pet food classification based on processing level should consider key factors including cooking or thermal processing, physical processing or particle size, processing of ingredients, and additives (Figure 2). We propose these factors not as an official regulatory definition at this time, but as candidates to guide a future consensus framework.

- **Cooking or Thermal Processing.** “Cooking” is an AAFCO Official Feed Term defined as “heating in the presence of moisture to alter chemical and/or physical characteristics or to sterilise (68).” This factor reflects the thermal processing applied to the diet as higher temperatures and longer durations contribute to a higher level of processing. For example, thermal processing could be classified based on the highest temperature reached and the duration for which that temperature is held.

- Ultra-high heat: Cooking reaches 121 °C or above to achieve sterilisation. The product is usually shelf-stable at room temperature after manufacturing.
- High heat: The highest temperature during manufacturing reaches at least 100 °C but remains below 121 °C.
- Low heat: The highest temperature during manufacturing achieves pasteurisation (for example 63–65 °C for >30 min, or 75 °C for >8 min (188)) but remains below 100 °C. Depending on the moisture content, the diet may or may not be required to be refrigerated or frozen after the manufacturing before feeding.
- No heat: No heat is intentionally applied and the diet does not achieve pasteurisation. Ingredients are kept frozen during manufacturing and thawed to be at room temperature or heated to slightly higher than room temperature when feeding. If home-prepared, ingredients may be at room temperature without being frozen during preparation.

- However, this example still fails to capture the full complexity of thermal processing. For example, freeze-dried pet food goes through multiple processing steps involving extended durations and high pressure, even though cooking temperatures remain relatively low throughout (Table 3). Additionally, the source of thermal energy should be taken into account. The same final cooking temperature can be achieved through different combinations of mechanical energy (such as friction and compression inside the extruder) and external heat (from steam or electrical heating), even though varying levels of mechanical energy can lead to final products with different textures and physical properties (189–191). Therefore, a consensus on the most appropriate way to quantify “cooking” is needed.
- **Physical Processing or Particle Size.** This factor reflects the level of physical processing applied to the diet, such as cutting, grinding, or extruding, which modifies the physical properties of the food, including particle size. Particle size has been shown to influence nutrient digestibility in dogs, but only when certain ingredients are used. Digestibility was impacted by feed particle size when maize and sorghum were included (192), while no differences were observed among diets containing rice, meat and bone meal, or poultry by-product meal (192–194). These observations add complexity to the assessment of this factor and its relationship to degree of processing.
- **Preprocessing of Ingredients.** This factor reflects the level of thermal and physical processing applied to individual ingredients before the diet itself is manufactured, such as animal derivatives, rendered meat meals, hydrolysed meat, and preprocessed grains. For example, two extruded dry diets may undergo identical cooking and processing, but one may use rendered meat meals and preprocessed grains with smaller particle sizes, while the other uses fresh meat and unprocessed grains with larger particle sizes. Unless the framework accounts for the preprocessing of the ingredients, both diets would be classified as equally processed.
- **Additives.** This factor captures additives present in the diet. In the NOVA classification (7), only the additives included for purposes other than fortification contribute to the higher level of processing. Using the additive categories under the existing European Union regulation (EC 1831/2003) (92), we suggest a similar approach, where only the presence of technological or sensory additives increases processing level, since these two additive categories are used with a purpose to aid the processing. However, variability in the effects of different additives may be observed even within these two categories, and it is common for labels not to report the amount of each additive per calorie content. These limitations add complexity to the consideration of this factor and its contribution to the degree of processing.

The review of these key factors also highlights additional questions that must be addressed in order to accurately establish a classification system based on processing level. First, the relative importance of the abovementioned factors is unclear. One may argue that a long list of additives might indicate a higher degree of processing than extensive thermal or physical processing. Additionally, unlike the NOVA classification where foods are clearly divided into four groups (195, 196), there is currently no evidence-based approach to establish definitive cut-offs for absolute processing categories in pet food. Most importantly, the current regulations of product labelling may render pet food classification impractical. Under EC 1831/2003 (92), while additives must be declared on the label, other compounds that do not fit into these regulated categories, items considered “processing aids,” or substances migrating to food from equipment or packaging do not need to be declared on the label. Moreover, according to the Code of Good Labelling Practice for Pet Food published by the European Pet Food Industry Federation (197), there is no obligation to declare additives with no legal maximum limit. Additives of the functional groups including preservatives, antioxidants, flavourings and colourants need not be declared by name but can be declared by only the respective functional group (e.g., appearing as “antioxidants” on the label). This applies even when the level of the additive exceeds the recommended maximum level (185). Likewise, the FDA regulates the use and declaration of additives in the US. Additives may be classified as Generally Recognised as Safe (GRAS), an approved additive, or a processing aid. The FDA identifies processing aids in pet foods as: substances added to a food during processing but removed in some manner before packaged in finished form; substances added to a food during processing and are converted into constituents normally present in the food and do not significantly increase the amount of the constituents naturally found in the food; and substances added to a food for their technical or functional effect in the processing but are present in the finished food at insignificant levels and do not have any technical or functional effect in that food (21 CFR Subchapter B Subpart G) (89). If an additive meets the definition of a processing aid, it is exempt from declaration on the end product’s ingredient statement (198). The authors strongly encourage a revision of labelling regulations for additives to enhance transparency. Improved labelling would not only support research into optimal inclusion levels, but also help build consumer trust and awareness. Food processing classification cannot be precise until all feed materials and additives are publicly declared.

Other factors, while not contributing to the level of food processing, have been reported to be associated with health outcomes in pets. Therefore, we suggest that these additional factors be considered when examining the relationship between food processing and health outcomes in pets, as they could confound this association:

- **Nutritional Completeness and Balance.** This factor categorises diets based on their ability to meet established nutritional requirements.

- Complete and Balanced: Meets or exceeds established nutrient requirements for the pet’s life stage. This category encompasses commercially available diets labelled as such, as well as home-prepared diets that are formulated by board-certified veterinary nutritionists.
- Incomplete or Imbalanced: Does not meet established nutritional standards.

- **Sources of Micronutrient Fortification.** Evidence suggests different forms of vitamins and minerals used for fortification purposes may have differential impact on health. For example, copper sulphate was shown to have significantly higher bioavailability than copper oxide in dogs (88). Sodium selenite and selenium yeast were shown to have differential bioavailability and impact on selenium status, gut microbiome, and immune function in other animal species (199, 200). Therefore, identical diets using different forms of fortification may result in differential outcomes.
- **Moisture Level.** While moisture level does not contribute to the processing level, it is an intrinsic characteristic of a diet. For example, wet, mildly cooked, and RMBDs typically have a moisture level of 70% or above, while the moisture level of extruded dry kibbles and baked foods generally remains below 10–12% (Table 3). Studies in both dogs and cats suggest that diets with differential moisture content but identical dry matter may have differential impact on food intake, nutrient digestibility, and risk of diseases (101, 201–206).
- **Sourcing and Handling.** This factor addresses ingredient sourcing and handling of the diet.

- Human Grade: “Human grade” is an AAFCO Official Feed Term defined as “every ingredient and the resulting product must be stored, handled, processed, and transported in a manner that is consistent and compliant with 21 CFR Part 117 and those applicable federal human food laws as required by ingredient, process, and/or facility type (68).” Even if ingredients used may be fit for human consumption, they may not be considered human grade if they do not meet this definition.
- Feed Grade: “Feed grade” is an AAFCO Official Feed Term defined as “material that has been determined to be safe, functional, and suitable for its intended use in animal food, and is handled and labelled appropriately, and conforms to the Federal Food, Drug, and Cosmetic Act unless otherwise expressly permitted by the appropriate state or federal agency (suitable use in animal feed) (68).” Feed grade ingredients may or may not be of different quality, include less common parts of animal products or meat and meat byproducts from diseased animals (although under AAFCO’s regulations, they must be processed in a manner that eliminates disease-causing microorganisms prior to becoming animal feed (207)), and may not be subject to the same safety standards as human grade ingredients. Processing methods may also differ for ingredients intended for pet food than human food (e.g., rendered meals).

Even without a classification system, researchers should not be discouraged from investigating the different formats of pet food. Since their invention, traditional dry and canned foods have become the default or habitual diet for the majority of pet cats and dogs in many developed countries (208, 209). Compared to these diets, much less research has been conducted on other formats, despite emerging evidence in pets suggesting that they may have a differential impact on health as described above. Research directly comparing more-processed and less-processed pet foods, or characterising the physiological and biochemical properties of these alternative food types, is warranted. Additionally, one may argue understanding the health impacts of processing is even more critical in pets, since they generally have smaller dietary variety and variation compared to humans – their diet is largely confined to what their caretakers provide and typically remains consistent on a daily basis. Consequently, both the positive and the negative effects of a diet are magnified when animals are constantly exposed to the same food or diet format.

In conclusion, we highlight the need to establish a pet food classification system based on processing level in this Perspective piece. This Perspective is intended to provide a scientific foundation for subsequent efforts to introduce multi-stakeholder consensus, including organisations that develop model regulations such as AAFCO and FEDIAF. The impact of pet food processing level on canine and feline health cannot be accurately assessed without an objective approach to classify pet foods. Similar to the NOVA classification (7), we propose a list of key factors that should be considered as candidates, but given the complexities, limitations, and acknowledged gaps in research, we are currently unable to develop an initial classification system. An objective and systematic classification would facilitate standardised descriptions and comparisons, enabling researchers to conduct more meaningful comparative studies, and empowering veterinary professionals and pet owners with more detailed information to make informed decisions about pet nutrition (210, 211). Future research should focus on refining food processing classification to accommodate the diversity of pet food formats, establishing standardised definitions, and validating its utility in clinical and research settings to create evidence-based and comprehensive feeding guidelines for the veterinary community and pet owners (195). Consensus within the veterinary and pet food science communities will be crucial for the successful implementation and adoption of a classification system.

## Data Availability

The original contributions presented in the study are included in the article/Supplementary material, further inquiries can be directed to the corresponding author/s.
