# Supplementary material for: Does the definition of human ultra-processed foods apply to dog and cat foods? A review of pet food processing techniques, their impact on health, and a call for pet food processing classification
Source: Front Vet Sci. 2026 Mar 4;13:1690420. doi: 10.3389/fvets.2026.1690420 (PMC12997800; doi:10.3389/fvets.2026.1690420)
Supplement: Supplementary file 1 [file Table_1.pdf]

Supplemental Table 1. Key studies showing the properties of ultra-processed foods (UPFs) that potentially explain their negative impact on human health through various pathways.

**Possible mechanism of UPFs**	**First author and year of publication**	**Species**	**Subject description**	**Experimental design & duration**	**Intervention/Exposure vs Control/Comparator**	**Key significant findings (p<0.05)**
Low satiety and promotion of caloric intake	Hall 2019 (1)	human	inpatient adults (n=20) with BMI of 27±1.5 kg/m^2	a cross-over randomised trial with 14 days of each diet intervention	diet with *ad libitum* intake of UPFs (~500 kcal/d) VS unprocessed diet<br><br>Diets were matched for presented calories, sugar, fat, fiber, and macronutrients.	Weight changes were highly correlated with energy intake, with participants gaining 0.9±0.3 kg during the ultra-processed diet and losing 0.9±0.3 kg during the unprocessed diet.
	Galdino-Silva 2024 (2)	human	overweight or obese adults as determined by BMI, waist circumference, and body fat (n=42)	a randomised controlled trial with one test meal	UPF meal (n=22) VS non-UPF meal (n=20)<br><br>The meals had similar amounts of kilocalories, macronutrients, fiber, and sodium, differing only in the classification between UPF and non-UPF.	Individuals consuming the UPF meal had a significantly faster intake rate (07:52 ± 3:00 vs. 11:07 ± 03:16 min), with less chewing and bites, and greater capacity to eat (39.68 ± 22.69 vs. 23.95 ± 18.92 mm).
	Edwin Thanarajah 2023 (3)	human	healthy normal weight adults (n=49) with BMI of 22.64±0.37 kg/m^2	a randomised, controlled trial with 8 weeks of the intervention	high fat/high sugar yogurt (n=26) VS low fat/low sugar yogurt (n=23)<br><br>Yogurts were matched	The high-fat/high-sugar intervention decreased the preference for low-fat food while increasing brain response to food and

					for calories (78-79 kcal/serving), and added on top of the regular diet.	associative learning independent of food cues or reward. These alterations were independent of changes in body weight and metabolic parameters, indicating a direct effect of high-fat, high-sugar foods on neurobehavioral adaptations that may increase the risk for overeating and weight gain.
Accelerated nutrient absorption rate	Silva 2021 (4)	human	pregnant women (n=42) with pre-existing diabetes mellitus with age ≥20 years at conception	a prospective cohort study examining the association between UPF consumption and gestational weight gain and blood glucose measure changes during pregnancy	higher UPF intakes VS lower UPF intakes	UPF consumption influenced glycemic control and total gestational weight gain. The increase of every 1 kcal in the calorie intake from UPFs in the third trimester increased glycated hemoglobin by 0.007%, raised 1-h postprandial glucose by 0.14 mg/dL, and added 0.11 kg to total gestational weight gain.
Undesirable circulating lipid profile	Rauber 2015 (5)	human	children of low socioeconomic status (n=345)	a prospective cohort study examining the association between	higher UPF intakes VS lower UPF intakes	UPF consumption at preschool age was a predictor of a higher

and inflammation				UPF consumption at preschool age (3-4 years) and circulating lipid profile from preschool to school age (7-8 years)		increase in total cholesterol and LDL cholesterol from preschool to school age. For every 1% increase in energy intake from UPFs, total cholesterol increased by 0.430 mg/dL and LDL cholesterol increased by 0.369 mg/dL.
	Millar 2024 (6)	human	middle-to older-aged adults randomly selected from a large primary care centre (n=1986)	a cross-sectional study examining the association between UPF consumption and circulating cholesterol and lipoprotein profiles (high-, low-, and very low-density lipoproteins)	higher UPF intakes VS lower UPF intakes	Higher UPF consumption was associated with a more pro-atherogenic, insulin-resistant metabolic profile. Specifically, it was associated with reduced HDL cholesterol concentrations, large LDL, total and medium HDL concentrations, smaller LDL and HDL size, and increased medium VLDL levels, small LDL and HDL concentrations, and higher lipoprotein insulin resistance scores, reflecting greater lipoprotein-related insulin resistance.

	Lane 2022 (7)	human	a sub-group of adults from the Melbourne Collaborative Cohort Study (n=2018)	a cross-sectional study examining the association between UPF consumption and a marker of inflammation (serum high-sensitivity C-reactive protein)	higher UPF intakes VS lower UPF intakes	Every 100 g increase in UPF intake was associated with a 4.0% increase in hsCRP concentration.
	Lopes 2019 (8)	human	a sub-group of adults from the Longitudinal Study of Adult Health (n=8468)	a cross-sectional study examining the association between UPF consumption and a marker of inflammation (serum C-reactive protein)	higher UPF intakes VS lower UPF intakes	Among women, the highest tertile of UPF intake was associated with mean CRP levels that were 14% higher than those of the lowest tertile, but the association disappeared after adjusting for BMI. No significant association was observed in men.
Gut microbial dysbiosis & increased gut permeability	Snelson 2021 (9)	rodent	male Sprague-Dawley rats, C57BL/6 mice, and *Lepr*$^{db/db}$ (diabetic) mice	rat experiment - a randomised controlled experiment with 3 groups (n=5-10 per group) for 24 weeks:<br>- control (unbaked diet)<br>- heat-treated (processed) diet<br>- heat-treated diet + drug<br><br>C57BL/6 mouse experiment - a	heat-treated diet VS unbaked diet	All three experiments indicated that the heat-treated diet caused increased intestinal permeability and increased plasma lipopolysaccharide, indicating a leaky gut.

				randomised controlled experiment with 2 groups (n=7-12 per group) for 24 weeks:<br>- control (unbaked diet)<br>- heat-treated (processed) diet<br><br>*Lepr*$^{db/db}$ mouse experiment - a randomised controlled experiment with 3 groups (n=8-16 per group) for 10 weeks:<br>- control (unbaked diet)<br>- heat-treated (processed) diet<br>- heat-treated (processed) diet + resistant starch		
	Squizani 2022 (10)	rat	male Wistar rats (n=28)	a randomised controlled experiment with 4 groups (n=7 per group):<br>- control diet<br>- control diet + Zn<br>- cafeteria diet<br>- cafeteria diet + Zn<br><br>20 weeks of dietary intervention with Zn given during weeks 16–20	cafeteria diet (higher UPFs) VS control diet	The cafeteria diet reduced colon crypt depth and altered microbiome composition including decreased Firmicutes and increased Bacteroidetes.<br><br>The cafeteria diet also reduced claudin-5 in cerebral cortex and hippocampal

						synaptophysin.
	de la Garza 2022 (11)	rat	male Wistar rats (n=10)	a randomised controlled experiment with 2 groups (n=5 per group) for 15 weeks:<br>- control diet<br>- cafeteria diet	cafeteria diet (higher UPFs) VS control diet	The cafeteria diet group gained more weight, had decreased gut microbiota α-diversity, increased Firmicutes/Bacteroidetes ratio, decreased Prevotella, increased Lactobacillus, Anaerotruncus, and Sutterella.
High levels of advanced-glycation endproducts (AGEs)	Nowotny 2018 (12)	human	individual ranging from those with healthy weight, overweight individuals, patients with diabetes mellitus, to chronic kidney disease	a review of 4 review papers<br>- Kellow 2013: 16 trials<br>- van Puyvelde 2014: 12 trials & observational studies<br>- Clarke 2016: 11 trials<br>- Baye 2017: 23 trials	higher AGE intakes VS lower AGE intakes	Depending on the subject characteristics and doses of AGE, inflammation markers, vascular function, circulating lipid profiles, insulin sensitivity were shown to be improved with lower AGE diets in certain subgroups.
Inclusion of food additives	Chassaing 2022 (13)	human	healthy adults (n=16)	a randomised, controlled trial with 11 days of dietary intervention	identical diets with carboxymethylcellulose (CMC 15 g/d, n=7) VS without CMC (n=9)	CMC consumption increased postprandial abdominal discomfort, reduced microbial evenness, reduced short-chain fatty acids and free amino acids.
	Chassaing 2015 (14)	mouse	wild type, *IL10*$^{-/-}$, and *TLR*$^{-/-}$ C57BL/6 mice	experiment 1 - a randomised controlled	water containing emulsifiers (CMC or	Emulsifier-treated mice had microbiota

				experiment with 3 groups (n=5 per group) for 12 weeks:<br>- plain water<br>- water containing 1% CMC<br>- water containing 1% P80<br><br>experiment 2 - a randomised controlled experiment with 3 groups (n=10 per group) for 8 weeks:<br>- plain water<br>- water containing 1% CMC<br>- water containing 1% P80	P80) VS plain water	encroachment, increased colonic mucosa-adherent bacteria, altered gut microbiome, increased levels of fecal lipopolysaccharide and flagellin, increased intestinal permeability, increased food intake, body weight, fasting glucose, spleen/fat pad weight.
	Swidsinski 2009 (15)	mouse	*IL10*$^{-/-}$ mice (n=13)	a randomised controlled experiment with 2 groups for 3 weeks:<br>- plain water<br>- water containing 2% CMC	water containing CMC (n=7) VS plain water (n=6)	CMC-treated mice had distended spaces between villi, increased luminal bacterial concentration, presence of bacteria between villi, increased bacterial contact and surface coverage in villi.
	Suez 2014 (16)	mouse	C57BL/6 mice	a randomised controlled experiment where donor mice were given drinking water	donor mice: artificial sweeteners VS glucose/sucrose	Artificial sweetener consumption induced glucose intolerance vs glucose or water. Fecal

				supplemented with artificial sweeteners (saccharin, sucralose, or aspartame) or glucose/sucrose controls up to 11 weeks, and germ-free recipient mice received a microbiome transplant from the donor mice	germ-free mice: microbiome transplant from sweetener-fed (n=12) VS glucose-fed mice (n=11)	microbiota transplantation from sweetener-fed mice to germ-free mice transferred the glucose intolerance phenotype. Artificial sweeteners altered microbiome composition and gene expression involved in metabolic pathways.
	Mossavar-Rahmani 2019 (17)	human	post-menopausal women (n=81714)	a prospective cohort study examining the association between artificially sweetened beverage intakes and cardiovascular disease and mortality outcomes	High (≥2 time/day) VS medium (5-7 time/week) VS low (1-4 time/week) VS non-consumers (<1 time/week) of artificially sweetened beverages	Compared to non-consumers, high consumers had higher risks of any stroke (HR=1.23), ischemic stroke (HR=1.31), coronary heart disease (HR=1.29), all-cause mortality (HR=1.16), small artery occlusion ischemic stroke (HR=1.81).
	Debras 2022 (18)	human	adults from the French NutriNet-Santé cohort (n=103388)	a prospective cohort study examining the association between artificial sweetener intakes (aspartame, acesulfame potassium, sucralose) and cardiovascular disease outcomes with a median	higher VS lower VS non-consumers of artificial sweeteners	Total artificial sweetener intake was associated with increased risk of cardiovascular disease (HR=1.09) and cerebrovascular disease (HR=1.18). Aspartame intake was significantly associated with

				follow-up of 9.0 years		cerebrovascular incidence (HR=1.17). Acesulfame potassium intake was significantly associated with coronary heart disease (HR=1.40).
	Debras 2023 (19)	human	adults from the French NutriNet-Santé cohort (n=105588)	a prospective cohort study examining the association between artificial sweetener intakes (aspartame, acesulfame potassium, sucralose) and incidence of type 2 diabetes with a median follow-up of 9.13 years	higher VS lower VS non-consumers of artificial sweeteners	Total artificial sweetener intake was associated with increased risk of type 2 diabetes (HR=1.69). Each sweetener was also significantly associated with the increased risk (aspartame HR=1.63, acesulfame potassium HR=1.70, sucralose HR=1.34)
	Said Abasse 2022 (20)	human	individuals with a wide range of characteristics	a meta-analysis examining the association between nitrate and nitrite intakes and cancer risks with 3 studies included for thyroid cancer and 4 studies included for glioma	highest VS lowest intake of nitrate and nitrite	When comparing highest to lowest (reference) categories of intake, meta-analysis of studies showed that higher nitrate intake was associated with an increased risk of thyroid cancer (OR = 1.40), and higher nitrite intake was associated with an increased risk of glioma (OR = 1.12).

	Chazelas 2022 (21)	human	adults from the French NutriNet-Santé cohort (n=101056)	a prospective cohort study examining the association between nitrite and nitrate intakes and cancer risk with a median follow-up of 6.7 years	high VS medium VS low nitrite and nitrate intakes	High consumers of food additive nitrates had higher breast cancer risk (HR=1.24), more specifically for potassium nitrate. High consumers of food additive nitrites had higher prostate cancer risk (HR=1.58), specifically for sodium nitrite.

References

1. Hall KD, Ayuketah A, Brychta R, Cai H, Cassimatis T, Chen KY, et al. Ultra-processed diets cause excess calorie intake and weight gain: An inpatient randomized controlled trial of ad libitum food intake. Cell Metab. 2019 Jul 2;30(1):67–77.e3.

2. Galdino-Silva MB, Almeida KMM, Oliveira ADS de, Santos JVLD, Macena M de L, Silva DR, et al. A meal with ultra-processed foods leads to a faster rate of intake and to a lesser decrease in the capacity to eat when compared to a similar, matched meal without ultra-processed foods. Nutrients. 2024 Dec 21;16(24):4398.

3. Edwin Thanarajah S, DiFeliceantonio AG, Albus K, Kuzmanovic B, Rigoux L, Iglesias S, et al. Habitual daily intake of a sweet and fatty snack modulates reward processing in humans. Cell Metab. 2023 Apr 4;35(4):571–84.e6.

4. Silva CFM, Saunders C, Peres W, Folino B, Kamel T, Dos Santos MS, et al. Effect of ultra-processed foods consumption on glycemic control and gestational weight gain in pregnant with pregestational diabetes mellitus using carbohydrate counting. PeerJ. 2021 Feb 1;9(e10514):e10514.

5. Rauber F, Campagnolo PDB, Hoffman DJ, Vitolo MR. Consumption of ultra-processed food products and its effects on children’s lipid profiles: a longitudinal study. Nutr Metab Cardiovasc Dis. 2015 Jan 1;25(1):116–22.

6. Millar SR, Harrington JM, Perry IJ, Phillips CM. Ultra-processed food and drink consumption and lipoprotein subclass profiles: A cross-sectional study of a middle-to older-aged population. Clin Nutr. 2024 Sep 1;43(9):1972–80.

7. Lane MM, Lotfaliany M, Forbes M, Loughman A, Rocks T, O'Neil A, et al. Higher ultra-processed food consumption is associated with greater high-sensitivity C-reactive protein concentration in adults: Cross-sectional results from the Melbourne Collaborative Cohort Study. Nutrients. 2022 Aug 12;14(16):3309.

8. Lopes AE da SC, Araújo LF, Levy RB, Barreto SM, Giatti L. Association between consumption of ultra-processed foods and serum C-reactive protein levels: cross-sectional results from the ELSA-Brasil study. Sao Paulo Med J. 2019 Jul 15;137(2):169–76.

9. Snelson M, Tan SM, Clarke RE, de Pasquale C, Thallas-Bonke V, Nguyen TV, et al. Processed foods drive intestinal barrier permeability and microvascular diseases. Sci Adv. 2021 Mar;7(14):eabe4841.

10. Squizani S, Jantsch J, Rodrigues F da S, Braga MF, Eller S, de Oliveira TF, et al. Zinc supplementation partially decreases the harmful effects of a cafeteria diet in rats but does not prevent intestinal dysbiosis. Nutrients. 2022 Sep 22;14(19):3921.

11. de la Garza AL, Martínez-Tamez AM, Mellado-Negrete A, Arjonilla-Becerra S, Peña-Vázquez GI, Marín-Obispo LM, et al. Characterization of the cafeteria diet as simulation of the human Western diet and its impact on the lipidomic profile and gut Microbiota in obese rats. Nutrients. 2022 Dec 24;15(1):86.

12. Nowotny K, Schröter D, Schreiner M, Grune T. Dietary advanced glycation end products and their relevance for human health. Ageing Res Rev. 2018 Nov 1;47:55–66.

13. Chassaing B, Compher C, Bonhomme B, Liu Q, Tian Y, Walters W, et al. Randomized controlled-feeding study of dietary emulsifier carboxymethylcellulose reveals detrimental impacts on the gut Microbiota and metabolome. Gastroenterology. 2022 Mar 1;162(3):743–56.

14. Chassaing B, Koren O, Goodrich JK, Poole AC, Srinivasan S, Ley RE, et al. Dietary emulsifiers impact the mouse gut microbiota promoting colitis and metabolic syndrome. Nature. 2015 Mar 5;519(7541):92–6.

15. Swidsinski A, Ung V, Sydora BC, Loening-Baucke V, Doerffel Y, Verstraelen H, et al. Bacterial overgrowth and inflammation of small intestine after carboxymethylcellulose ingestion in genetically susceptible mice. Inflamm Bowel Dis. 2009 Mar;15(3):359–64.

16. Suez J, Korem T, Zeevi D, Zilberman-Schapira G, Thaiss CA, Maza O, et al. Artificial sweeteners induce glucose intolerance by altering the gut microbiota. Nature. 2014 Oct 9;514(7521):181–6.

17. Mossavar-Rahmani Y, Kamensky V, Manson JE, Silver B, Rapp SR, Haring B, et al. Artificially sweetened beverages and stroke, coronary heart disease, and all-cause mortality in the women's health initiative. Stroke. 2019 Mar;50(3):555–62.

18. Debras C, Chazelas E, Sellem L, Porcher R, Druesne-Pecollo N, Esseddik Y, et al. Artificial sweeteners and risk of cardiovascular diseases: results from the prospective NutriNet-Santé cohort. BMJ. 2022 Sep 7;378:e071204.

19. Debras C, Deschasaux-Tanguy M, Chazelas E, Sellem L, Druesne-Pecollo N, Esseddik Y, et al. Artificial sweeteners and risk of type 2 diabetes in the prospective NutriNet-Santé cohort. Diabetes Care. 2023 Sep 1;46(9):1681–90.

20. Said Abasse K, Essien EE, Abbas M, Yu X, Xie W, Sun J, et al. Association between dietary nitrate, nitrite intake, and site-specific cancer risk: A systematic review and meta-analysis. Nutrients. 2022 Feb 4;14(3):666.

21. Chazelas E, Pierre F, Druesne-Pecollo N, Esseddik Y, Szabo de Edelenyi F, Agaesse C, et al. Nitrites and nitrates from food additives and natural sources and cancer risk: results from the NutriNet-Santé cohort. Int J Epidemiol. 2022 Aug 10;51(4):1106–19.
